# Fucoxanthin Attenuates Free Fatty Acid-Induced Nonalcoholic Fatty Liver Disease by Regulating Lipid Metabolism/Oxidative Stress/Inflammation via the AMPK/Nrf2/TLR4 Signaling Pathway

**DOI:** 10.3390/md20040225

**Published:** 2022-03-25

**Authors:** Jiena Ye, Jiawen Zheng, Xiaoxiao Tian, Baogui Xu, Falei Yuan, Bin Wang, Zuisu Yang, Fangfang Huang

**Affiliations:** Zhejiang Provincial Engineering Technology Research Center of Marine Biomedical Products, School of Food and Pharmacy, Zhejiang Ocean University, Zhoushan 316022, China; z20105500018@zjou.edu.cn (J.Y.); jwzheng1996@163.com (J.Z.); z18095135044@zjou.edu.cn (X.T.); Z19105500022@ziou.edu.cn (B.X.); yuanfalei@zjou.edu.cn (F.Y.); wangbin@zjou.edu.cn (B.W.)

**Keywords:** NAFLD, fucoxanthin, adenosine monophosphate-activated protein kinase (AMPK), nuclear factor erythroid-2-related factor 2-mediated (Nrf2), Toll-like receptor 4-mediated (TLR4)

## Abstract

Fucoxanthin, a xanthophyll carotenoid abundant in brown algae, is reported to have several biological functions, such as antioxidant, anti-inflammatory, and anti-tumor activities, in mice. We investigated the effects and mechanisms of fucoxanthin in the mixture oleate/palmitate = 2/1(FFA)-induced nonalcoholic fatty liver disease (NAFLD) cell model in this study. The results showed that the content of superoxide dismutase in the FFA group was 9.8 ± 1.0 U/mgprot, while that in the fucoxanthin high-dose (H-Fx) group (2 μg/mL) increased to 22.9 ± 0.6 U/mgprot. The content of interleukin-1β in the FFA group was 89.3 ± 3.6 ng/mL, while that in the H-Fx group was reduced to 53.8 ± 2.8 ng/mL. The above results indicate that fucoxanthin could alleviate the FFA-induced oxidative stress and inflammatory levels in the liver cells. Oil red-O staining revealed visible protrusions and a significant decrease in the number of lipid droplets in the cytoplasm of cells in the fucoxanthin group. These findings on the mechanisms of action suggest that fucoxanthin can repair FFA-induced NAFLD via the adenosine monophosphate-activated protein kinase (AMPK) signaling pathway and nuclear factor erythroid-2-related factor 2-mediated (Nrf2) signaling pathway, as well as by downregulating the expression of the Toll-like receptor 4-mediated (TLR4) signaling pathway. Fucoxanthin exhibited alleviating effects in the FFA-induced NAFLD model and could be explored as a potential anti-NAFLD substance.

## 1. Introduction

Nonalcoholic fatty liver disease (NAFLD), a heterogeneous disease with highly variable molecular mechanisms, has a high prevalence and is the most common chronic liver disease in the world [1]. The liver metabolizes lipids and stores triglycerides (TG) derived from the conversion of excess fatty acids [2]. Fatty liver disease, a condition in which the amount of TG accounts for more than 5% of the weight of the liver, impairs organ function. The accumulation of TG in the liver is thought to be an adaptive response to compensate for the increased cellular content of free fatty acids (FFAs), thereby preventing the onset of hepatocellular damage [3]. The increasing prevalence of factors such as obesity, diabetes, and inflammation, which are associated with NAFLD, has sparked growing interest in the disease [4].

NAFLD encompasses multiple damaging “hits” [5], such as insulin resistance, which lead to disorders of the lipid metabolism, and oxidative stress, which is an inflammatory response induced by reactive oxygen species. Lipid peroxidation reactions can induce and amplify cellular damage, mainly due to the formation of oxidation products generated by the reaction. Recent evidence suggests that hepatic accumulation of excess FFAs promotes increased β-oxidation, which ultimately leads to oxidative stress [6,7]. FFAs activate Kupffer cells through indirect stimulation of the toll-like receptor 4-mediated (TLR4)-mediated signaling pathway, leading them to adopt a pro-inflammatory phenotype [8]. Recent evidence suggests that activation of the TLR4 signaling pathway helps reduce palmitate-induced hepatotoxicity. *Gynostemma pentaphyllum* can improve the level of inflammatory factors in the NAFLD model by regulating the TLR4 signaling pathway [9,10]. Adenosine monophosphate-activated protein kinase (AMPK) is mainly expressed in the liver and skeletal muscle, and regulates lipid metabolism. Kangtaizhi granules, a Chinese medicine compound, effectively ameliorate the mixture of oleate/palmitate = 2/1(FFA)-induced lipid accumulation in HepG2 cells via the AMPK signaling pathway, thereby preventing nonalcoholic steatosis [11]. M3G, one of the main anthocyanins in blueberries, acts on the FFA-induced NAFLD cell model by activating the Nrf2/ARE (antioxidant response element) signaling pathway [12].

Fucoxanthin, an orange-red carotenoid, can be isolated from brown algae [13]. Fucoxanthin exhibits a strong antioxidant activity by inhibiting 2,2-Diphenyl-1-picrylhydrazyl (DPPH) and 2,2′-Azinobis-(3-ethylbenzthiazoline-6-sulphonate) (ABTS) radicals, and alleviates obesity by reducing lipogenesis [14,15]. Fucoxanthin also has anti-inflammatory, anti-bacterial, and anti-cancer properties [16,17]. Fucoxanthin not only improves lipid metabolism, but also attenuates mitochondrial dysfunction. Fucoxanthin alleviates palmitate-induced inflammatory responses in RAW 264.7 cells [18]. In addition to inhibiting hepatic oxidative stress and inflammation, fucoxanthin prevents the early phase of fibrosis in diet-induced nonalcoholic steatohepatitis in mice [19]. Potential protective functions of fucoxanthin against the development of NAFLD have been recognized [20,21]. Low molecular weight fucoidan and high stability fucoxanthin ameliorate hepatic steatosis, inflammation, fibrosis, and insulin resistance in NAFLD patients [22]. Fucoxanthin significantly inhibits the expression of sterol regulatory element binding proteins-1c (SREBP-1c) and decreases the expression of fatty acid synthase (FAS), phosphorylated AMPK, and phosphorylated acetyl-CoA carboxylase (ACC) in oleic acid-induced FL83B cells [23]. Nitzschia laevis extract prevents NAFLD by significantly inhibiting lipid accumulation in HepG2 cells, and increasing the gene and protein expression of phosphorylated ACC [24]. Recent studies found that fucoxanthin also enhances the expression of heme oxygenase-1 (HO-1) and nicotinamide quinone oxidoreductase 1 (NQO1), leading to antioxidant effects in murine hepatic BNL CL.2 cells [25]. Fucoxanthin enhances the antioxidant response mediated by nuclear factor-erythroid factor 2-related factor 2 (Nrf2), and inhibition of TLR4-induced signaling pathways is associated with the prevention of liver inflammation [26]. Whether the reparative effects of fucoxanthin on the FFA-induced NAFLD cell model are related to FAS, SREBP-1c protein, Nrf2, and TLR4 pathways, however, remains unclear. In this study, we used FFA-induced nonalcoholic fatty liver cells as a model and investigated whether fucoxanthin regulated AMPK, Nrf2, and TLR4 pathways in FFA-induced NAFLD.

## 2. Results

### 2.1. Determination of the Dosing Concentration

**Figure 1:**
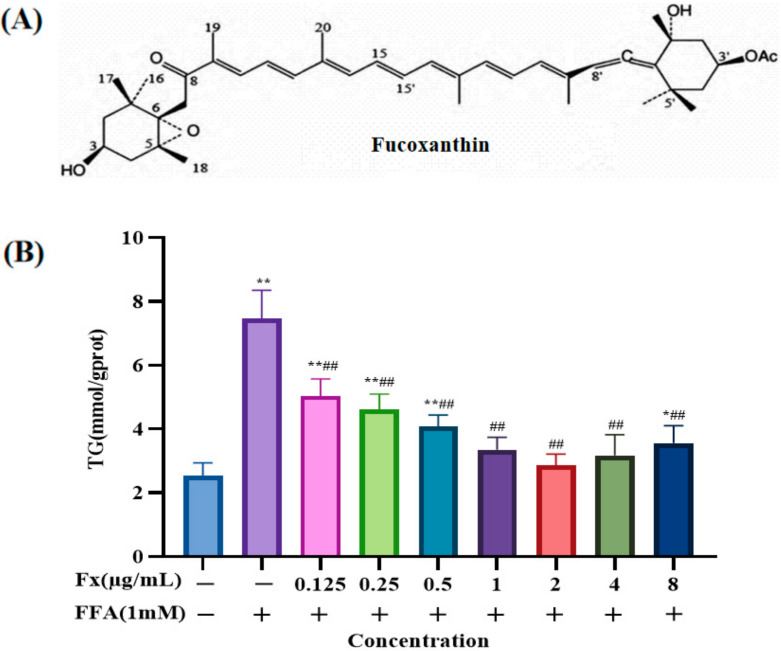
(**A**) Chemical structure of fucoxanthin. (**B**) Effect of different concentrations of fucoxanthin on the levels of triglycerides (TG) in FFA-treated liver cells. Data are expressed as mean ± standard deviation (*n* = 3), * *p* < 0.05, ** *p* < 0.01 vs. control group, ## *p* < 0.01 vs. FFA group.

The TG content in the cells of each group was measured under treatment with different concentrations of fucoxanthin after FFA induction for 24 h. The TG content was significantly higher in the FFA group (7.5 ± 0.7 mmol/gprot) than that in the control group (2.5 ± 0.3 mmol/gprot) (Figure 1B) (*p* < 0.01), demonstrating that the NAFLD cell model was successfully constructed. Establishing useful cellular models of lipid accumulation will significantly contribute to studies of the mechanisms of NAFLD, as well as its effective prevention and treatment [3]. Due to the short study period and reproducibility, in vitro cellular models of lipid accumulation have become a major approach for exploring this disease. Saturated or unsaturated fatty acid stimulation, causing intracellular lipid accumulation, is commonly used to construct NAFLD cell models [27,28]. FFA, consisting of oleic and palmitic acids [27], acts on the liver cells to cause intracellular lipid accumulation. FFA modeling methods can simulate the pathogenic process of NAFLD caused by disorders of the lipid metabolism, thereby facilitating studies of the specific mechanism of NAFLD and contributing to the development of novel therapeutic approaches [29]. The results of this study show that fucoxanthin intervention alone significantly reduced the NAFLD cell model TG level. When the concentration of fucoxanthin was 2 μg/mL, the treatment was the most effective. Therefore, the doses of 0.5 µg/mL (L-Fx group), a medium dose of 1 µg/mL (M-Fx group), and a high dose of 2 µg/mL (H-Fx group) were chosen to conduct the subsequent experiments.

### 2.2. Effect of Fucoxanthin on Intracellular Alanine Transaminase (ALT) and Aspartate Transaminase (AST) Viability

**Figure 2:**
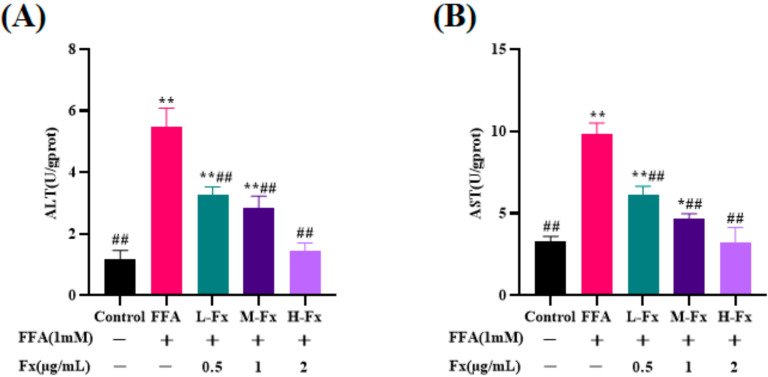
Effects of fucoxanthin on the levels of alanine transaminase (ALT) (**A**) and aspartate transaminase (AST) (**B**) activities in FFA-treated liver cells. Data are expressed as mean ± standard deviation (*n* = 3), * *p* < 0.05, ** *p* < 0.01 vs. control group, ## *p* < 0.01 vs. FFA group.

ALT and AST levels are important indicators of hepatocyte injury. When severe necrosis occurs in hepatocytes, the permeability of cell membranes increases, causing ALT and AST to enter the bloodstream, and the hepatocyte injury will increase significantly. The ALT and AST in FFA-induced liver cells were 5.5 ± 0.5 U/gprot and 9.8 ± 0.6 U/gprot, respectively. After fucoxanthin treatment, both ALT and AST were significantly reduced, and the expression level dropped to 1.4 ± 0.2 U/gprot and 3.3 ± 0.7 U/gprot, respectively (Figure 2) (*p* < 0.01). ALT and AST levels, as sensitive indicators of hepatocyte injury, increased significantly after FFA treatment, but reduced to different degrees after treatment with fucoxanthin. These findings demonstrated that fucoxanthin has significant reparative effects against the cell damage caused by FFA.

### 2.3. Effect of Fucoxanthin on Intracellular Lipid Accumulation

**Figure 3:**
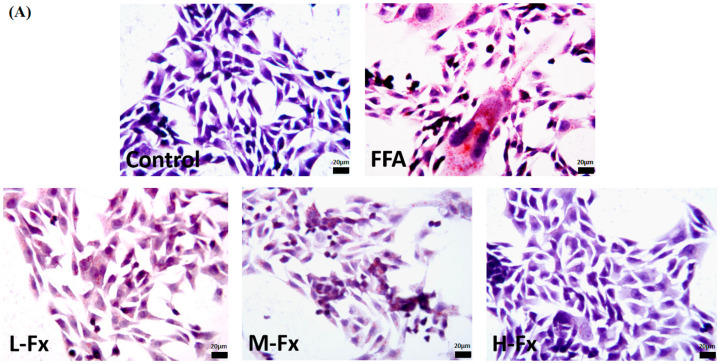

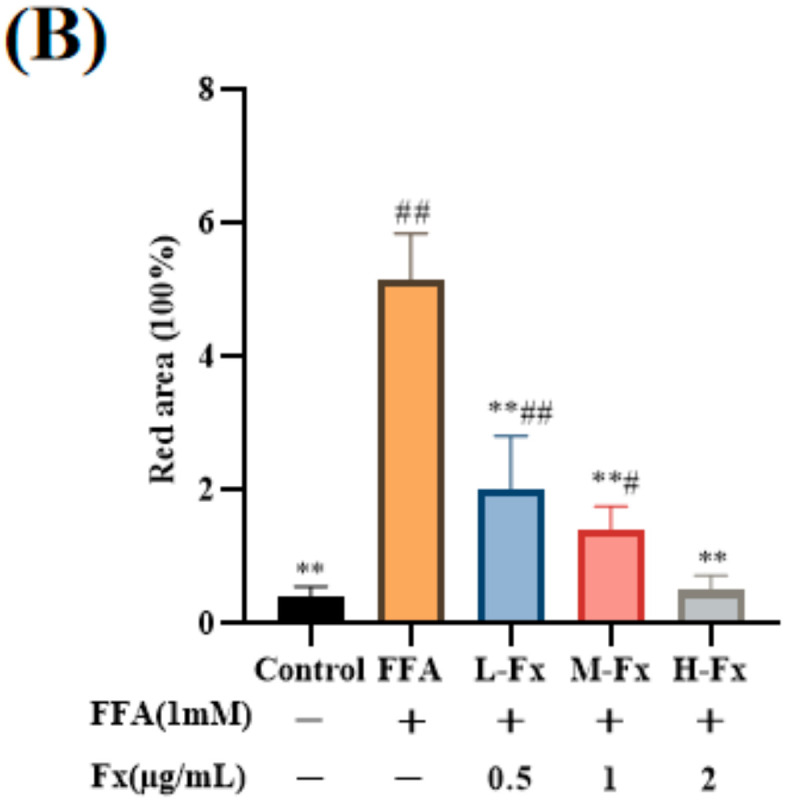
Oil Red O staining of FFA-treated liver cells (×400, scale bars of images are 20 μm) (**A**) and oil droplets analyzed by Image J (**B**). Data are expressed as mean ± standard deviation (*n* = 5), ** *p* < 0.01 vs. control group, # *p* < 0.05, ## *p* < 0.01 vs. FFA group.

The distribution of cellular lipid droplets and the morphologic changes of cells in each experimental group were observed by Oil Red O staining, and the parts of the cytoplasm with red represented lipid droplets (Figure 3). The cells of the control group were tightly connected and had relatively clear edges. At the same time, there were protrusions and good extensions around the cells. It can also be observed that the lipid droplet content in the cytoplasm is relatively low, and the nuclei were clearly visible. On the contrary, FFA-treated cells showed obvious lipid accumulation, reduced cell protrusions, partially solidified nuclei, obvious changes in the morphology of some cells, and an obvious increase of lipid droplets in the cytoplasm, with a large amount of lipid deposition, indicating that hepatocytes were damaged (Figure 3B). Lipid droplets in the cytoplasm of the fucoxanthin group was significantly decreased with the concentration ranging from 0.5 to 2 µg/mL, indicating that fucoxanthin could improve FFA-induced hepatic steatosis.

### 2.4. Effect of Fucoxanthin on Intracellular TG and Total Cholesterol (TC) Content

**Figure 4:**
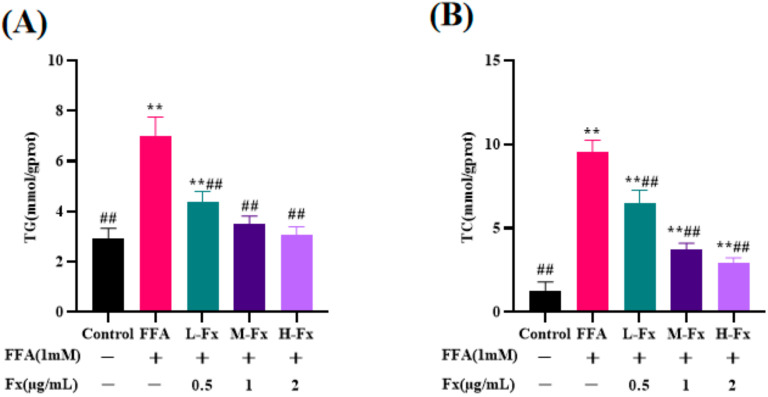
Effects of fucoxanthin on the levels of TG (**A**) and total cholesterol (TC) (**B**) in the FFA-treated liver cells. Data are expressed as mean ± standard deviation (*n* = 3), ** *p* < 0.01 vs. control group, ## *p* < 0.01 vs. FFA group.

Compared with the control group, the TC and TG contents were increased in the FFA-induced hepatocyte steatosis group (Figure 4) (*p* < 0.01), indicating that FFA induction leads to lipid accumulation. In contrast to the FFA-treated group, the fucoxanthin group had a significantly reduced TC and TG content, and this finding was consistent with the results of Oil Red O staining. These phenomena were significantly improved by treatment with fucoxanthin, indicating that fucoxanthin could effectively attenuate FFA-induced lipid deposition in the liver cells.

### 2.5. Effect of Fucoxanthin on Intracellular Antioxidant Enzyme Activity

**Figure 5:**
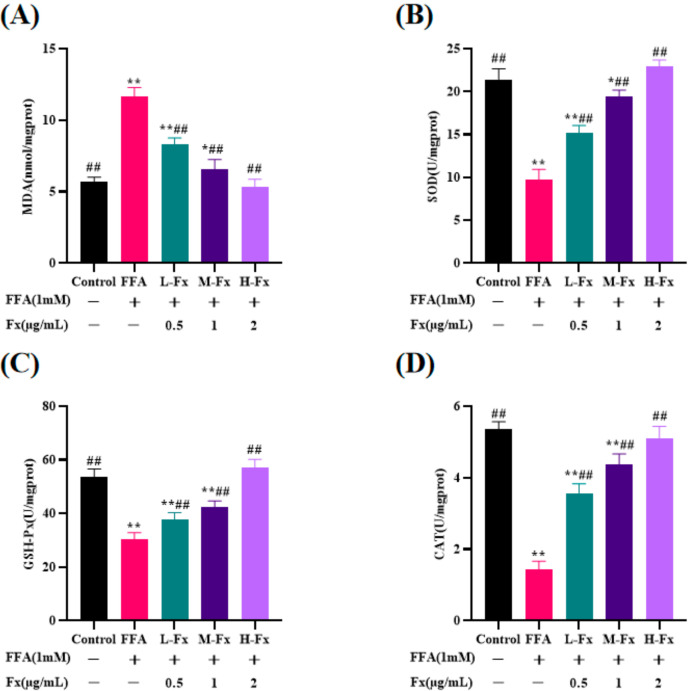
Effects of fucoxanthin on the levels of malondialdehyde (MDA) (**A**), superoxide dismutase (SOD) (**B**), glutathione peroxidase (GSH-Px) (**C**), and catalase (CAT) (**D**) in FFA-treated liver cells. Data are expressed as mean ± standard deviation (*n* = 3), * *p* < 0.05, ** *p* < 0.01 vs. control group, ## *p* < 0.01 vs. FFA group.

Lipid peroxidation in the cells was assessed by measuring the intracellular malondialdehyde (MDA) content and the activity of antioxidant enzymes, including superoxide dismutase (SOD), glutathione peroxidase (GSH-Px) and catalase (CAT). After incubating with FFA, the MDA content was dramatically higher in the cells than in the Control group, while the activity of the antioxidant enzymes (SOD, GSH-Px, and CAT) was remarkably reduced (Figure 5) (*p* < 0.01). The cellular MDA level decreased from 11.7 ± 0.5 nmol/mgprot in the FFA group to 5.3 ± 0.5 nmol/mgprot in the H-Fx group, suggesting that fucoxanthin improves in-cell lipid peroxidation in the cells induced by FFA.

### 2.6. Effect of Fucoxanthin on Intracellular Pro-Inflammatory Cytokines Content

**Figure 6:**
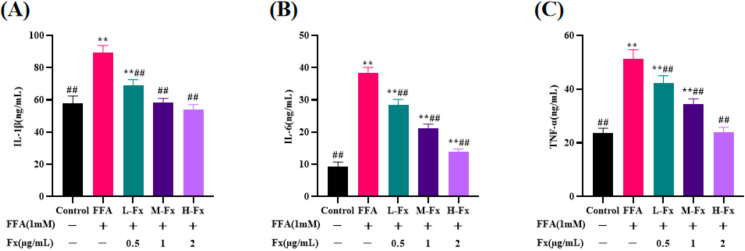
Effects of fucoxanthin on the levels of interleukin (IL)-1β (**A**), IL-6 (**B**) and tumor necrosis factor-α (TNF-α) (**C**) in FFA-treated liver cells. Data are expressed as mean ± standard deviation (*n* = 3), ** *p* < 0.01 vs. control group, ## *p* < 0.01 vs. FFA group.

Previous studies have demonstrated that progressive NAFLD could lead to an inflammatory response, causing the release of pro-inflammatory factors [30]. Therefore, the secretion levels of interleukin (IL)-6, IL-1β, and tumor necrosis factor-α (TNF-α) in the cell culture medium were measured with an enzyme-linked immunosorbent assay. The expressions of IL-6, IL-1β, and TNF-α were partly regulated in FFA-induced cells. The expressions of IL-6, IL-1β, and TNF-α proteins were significantly increased in FFA-induced the liver cells compared with the control group (Figure 6) (*p* < 0.01). A marked decrease in the expression of IL-6, IL-1β, and TNF-α protein was observed following treatment with fucoxanthin.

### 2.7. Effects of AMPK Activation and Expression of Genes Related to Lipid Metabolism

**Figure 7:**
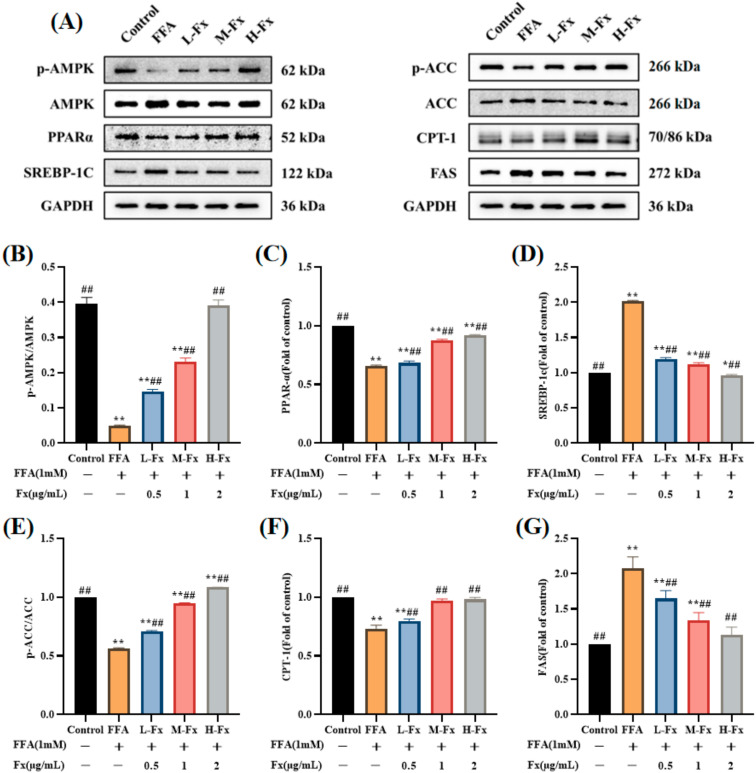
Effects of adenosine monophosphate-activated protein kinase (AMPK) activation and expression of genes related to lipid metabolism. (**A**) Western blot analysis of p-AMPK, AMPK, sterol regulatory element binding proteins-1c (SREBP-1c) and peroxisome proliferator-activated receptor α (PPARα), phosphorylated acetyl-CoA carboxylase (ACC) and ACC, carnitine palmitoyltransferase-1 (CPT-1), and fatty acid synthase (FAS) in the FFA-treated cells; (**B**) quantitative analysis for p-AMPK/AMPK; (**C**) quantitative analysis for PPARα; (**D**) quantitative analysis for SREBP-1c; (**E**) quantitative analysis for p-ACC/ACC; (**F**) quantitative analysis for CPT-1; (**G**) quantitative analysis for FAS. Data are expressed as mean ± standard deviation (*n* = 3), * *p* < 0.05, ** *p* < 0.01 vs. control group, ## *p* < 0.01 vs. FFA group.

Lipid metabolism in the liver is influenced by the balanced relationship between the lipid synthesis pathway and the β-oxidation pathway. To elucidate the mechanism of fucoxanthin to inhibit FFA-induced lipid accumulation in hepatocytes, the expression levels of the AMPK pathway-related proteins were examined (Figure 7) (*p* < 0.01). Activation of the AMPK protein both promotes fatty acid oxidation and inhibits lipid and cholesterol synthesis. Figure 7 shows that the significant reduction of phosphorylated AMPK levels in the FFA group was reversed by fucoxanthin. SREBP-1c and peroxisome proliferator-activated receptor α (PPARα) are important transcription factors that regulate the lipid metabolism in hepatocytes, and fucoxanthin regulates lipid metabolism in the liver by activating AMPK, thereby upregulating PPARα and downregulating the expression of SREBP-1c. FFA induction affects the expression of ACC and FAS, proteins related to lipid synthesis, and the results show that the phosphorylated ACC expression decreased and FAS levels increased in the FFA group, while the fucoxanthin group showed a recovery of the expression of both proteins. Carnitine palmitoyltransferase-1 (CPT-1) is a key enzyme involved in fatty acid oxidation, FFA induces a decrease in the level of CPT-1, and fucoxanthin treatment can reverse the above changes, indicating that fucoxanthin could improve hepatic steatosis by increasing fatty acid oxidation.

### 2.8. Effects of Fucoxanthin on the Nrf2-Mediated Antioxidant Response

**Figure 8:**
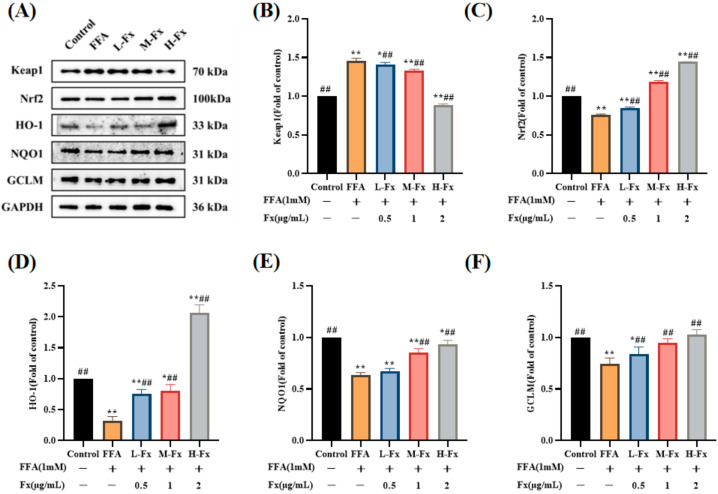
Effects of fucoxanthin on the nuclear factor erythroid-2-related factor 2-mediated (Nrf2) antioxidant response. (**A**) western blot analysis of kelch-like ECH-associated protein 1 (Keap-1), Nrf2, heme oxygenase-1 (HO-1), nicotinamide quinone oxidoreductase 1 (NQO1) and glutamate-cysteine ligase modifier subunit (GCLM) in the FFA-treated cells; (**B**) quantitative analysis for Keap-1; (**C**) quantitative analysis for Nrf2; (**D**) quantitative analysis for HO-1; (**E**) quantitative analysis for NQO1; (**F**) quantitative analysis for GCLM. Data are expressed as mean ± standard deviation (*n* = 3), * *p* < 0.05, ** *p* < 0.01 vs. control group, ## *p* < 0.01 vs. FFA group.

A recent study showed that liraglutide has a protective effect against FFA-induced oxidative stress, possibly via modulation of the Nrf2 pathway in the liver cells [31]. To further clarify the mechanism underlying the effect of fucoxanthin on FFA-induced intracellular lipid peroxidation, the expression of Nrf2 and its client proteins was examined. The expression of the Kelch-like ECH-associated protein 1 (Keap-1) protein was increased in the FFA group, and the expression of the Nrf2 protein was remarkably reduced (Figure 8) (*p* < 0.01). In contrast, the Keap-1 protein level was downregulated after treatment with fucoxanthin, while the expression of the Nrf2 protein was significantly restored. In addition, the expression levels of the downstream antioxidant proteins HO-1, NQO1, and glutamate-cysteine ligase modifier subunit (GCLM), which are associated with Nrf2 protein expression, were also significantly reversed. These results indicate that fucoxanthin reduces oxidative stress in hepatocytes by activating the Nrf2-mediated antioxidant response.

### 2.9. Effect of Fucoxanthin on the TLR4-Induced Inflammatory Response

**Figure 9:**
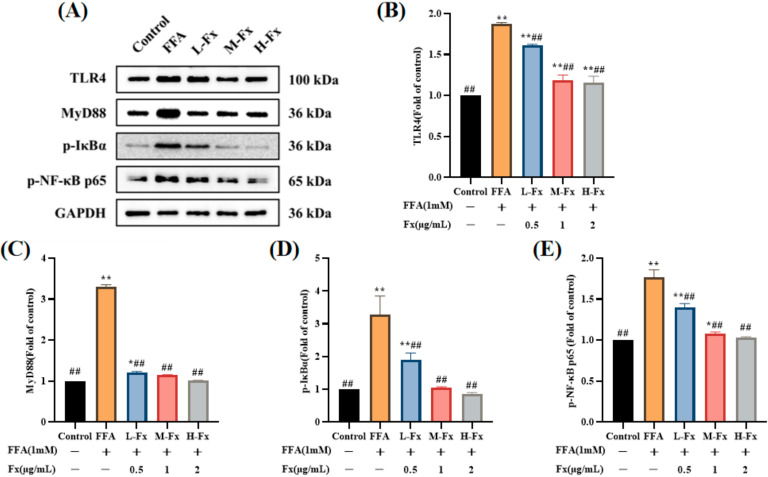
Effects of fucoxanthin on the toll-like receptor 4-mediated (TLR4)-induced inflammatory response. (**A**) Western blot analysis of TLR4, myeloid differentiation factor 88 (MyD88), and p-IκBα and p-NF-κB p65 in the FFA-treated cells; (**B**) the protein levels of TLR4; (**C**) the protein levels of MyD88; (**D**) the protein levels of p-IκBα; (**E**) the protein levels of p-NF-κB p65. Data are expressed as mean ± standard deviation (*n* = 3), * *p* < 0.05, ** *p* < 0.01 vs. control group, ## *p* < 0.01 vs. FFA group.

The protein expression of the TLR4 signaling pathway in each group of the liver cells is shown in Figure 9. The expression of TLR4 and its downstream proteins myeloid differentiation factor 88 (MyD88), p-IκBα, and p-NF-κBp65 was significantly upregulated in the FFA group, and was significantly inhibited by treatment with fucoxanthin. Research has shown that feprazone prevented FFA-induced activation of the TLR4/MyD88/NF-κB signaling pathway [32]. These results suggest that the TLR4 signaling pathway could be a target in anti-inflammation and lipid metabolism disorders.

## 3. Discussion

In this study, the intracellular TG content was used for screening to determine the optimal treatment concentration of fucoxanthin. Our results demonstrated that the TG and TC contents in the liver cells were markedly increased after the induction with FFA, and the results of Oil Red O staining showed that the cells underwent steatosis. These phenomena were significantly improved by treatment with fucoxanthin, indicating that fucoxanthin improves FFA-induced lipid deposition in the liver cells. ALT and AST levels as sensitive indicators of hepatocyte injury were significantly increased after FFA induction in the liver cells, and were reduced to different degrees after treatment with fucoxanthin. These findings demonstrated that fucoxanthin has significant reparative effects against liver cell damage caused by FFA (*p* < 0.01). In addition, inflammatory factor levels were significantly increased in the FFA-induced hepatocyte medium, and the release of inflammatory factors IL-1β, IL-6, and TNF-α was considerably reduced by treatment with fucoxanthin (*p* < 0.01). Western blotting results demonstrated that the expressions of TLR4, MyD88, p-IκBα, and p-NF-κBp65 in the FFA group were significantly increased (*p* < 0.01), and the protein expressions of MyD88 and p-IκBα increased more than three-fold. There was no significant difference between the fucoxanthin treatment and the normal group. These results revealed that fucoxanthin has a repairing effect on FFA-induced liver cells by reducing inflammation.

AMPK acts as an energy receptor that systematically regulates energy homeostasis, and is therefore considered a potential target for the treatment of metabolic syndrome [33]. AMPK activity is reduced in the adipose tissue of obese animals and humans, and the restriction of the nutritional intake further increases AMPK activity, thus slowing the progression of obesity [34]. Previous studies have revealed that fucoxanthin regulates gluconeogenesis by activating AMPK in diabetic and obese mouse models [35,36]. We also reported that fucoxanthin increases the AMPK phosphorylation level, which is the main kinase regulator of the downstream lipogenic enzymes ACC, SREBP-1c, and FAS [37,38]. Among them, ACC is mainly found in the cytoplasm of tissues where lipids are synthesized [39]. In the liver, ACC catalyzes the formation of malonyl coenzyme A, which synthesizes long-chain fatty acid precursors [40]. SREBP-1c, an important transcription factor, is a key enzyme in the regulation of fatty acid synthesis [41], and FAS is a key enzyme of de novo fatty acid synthesis [42]. In the present research, we found that fucoxanthin could reduce lipid production by activating AMPK, thus inhibiting the expression of ACC, SREBP-1c, and FAS—key genes involved in lipid synthesis.

**Figure 10:**
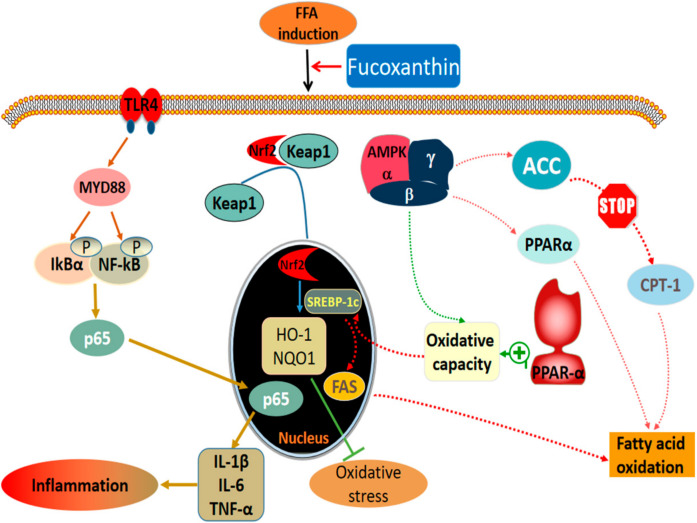
Mechanisms of fucoxanthin on alleviating NAFLD.

The PPARα expression is reduced in patients with NAFLD, compared with a high expression in normal liver [43]. Activation of PPARα induces the expression of fatty acid oxidation and export-related genes, and participates in the regulation of liver lipid metabolism by reducing liver lipid deposition [44,45]. CPT-1 is a key enzyme involved in fatty acid oxidation. Studies have found that PPARα can also increase fatty acid oxidation by inducing CPT-1 expression [46]. We found that FFA induced an increase in the amount of MDA, a lipid peroxide marker, and a decrease in activities of the antioxidant enzymes SOD, GSH-Px, and CAT, as well as decrease in the PPARα and CPT-1 expression levels. The imbalance of the liver oxidation-antioxidant system is caused by the accumulation of free radicals causing lipid peroxidation [47]. Fucoxanthin, however, increased the expression of PPARα and CPT-1, and decreased the MDA content. Meanwhile, fucoxanthin increased SOD, GSH-Px, and CAT activities and restored the expression of Nrf2-mediated antioxidant pathway-related proteins, thus regulating the cellular antioxidant system (Figure 10). The results suggest that PPARα is another effective target of fucoxanthin to improve lipid deposition in hepatocytes, and can improve the balance of cellular lipid metabolism through the PPARα pathway, thus reducing hepatocyte lipid accumulation. The structure of fucoxanthin contains propadiene, containing allene bond, 5,6-epoxy group, conjugated polyene chain, and other structures (Figure 1A); therefore, it has strong antioxidant properties. However, our work also has shortcomings, for example the fucoxanthin we provided was of low purity, and we should improve the purity of fucoxanthin and explore what these substances are.

## 4. Materials and Methods

### 4.1. Chemicals and Reagents

Fucoxanthin with a purity of 70% was purchased by Shandong Jiejing Group Corporation (Rizhao, China). AST, ALT, TC, TG, SOD, CAT, GSH-Px, MDA, and Oil Red O staining solution kits were all obtained from the Jiancheng Bioengineering Institute (Nanjing, China). Enzyme-linked immunosorbent assay (ELISA) kits and a bicinchoninic acid protein assay kit were obtained from BOSTER (Wuhan, China). AMPK, phosphorylated AMPK, PPARα, SREBP-1c, ACC, phosphorylated ACC, FAS, and CPT-1 antibodies were on sale at Affinity Biosciences, Inc. (Cincinnati, OH, USA). HO-1, Keap-1, GCLM, Nrf2, NQO1, GAPDH, MyD88, TLR4, phosphorylated IκBα, and phosphorylated NF-κBp65 antibodies were obtained from Wuhan Sanying Biotechnology Co. (Wuhan, China).

### 4.2. Cell Culture

Normal human Chang liver cells, purchased from the Cell Center of Xiangya School of Medicine, Central South University, were cultured in our laboratory for passaging. The thawed cells were transferred to 10 mL centrifuge tubes under an aseptic environment and centrifuged with 4 mL Dulbecco’s modified Eagle’s medium high sugar medium, the supernatant was discarded and the cell precipitate was left, and then 5–6 mL of the culture medium was added and gently blown to make it evenly dispersed into a single cell suspension, which was placed at 37 °C in a 5% CO_2_ incubator.

### 4.3. Establishment of NAFLD Cell Model

The NAFLD cell model was established by referring to the method of Qing Xiao et al. [48]. We dissolved oleic acid and palmitic acid with sodium hydroxide, and mixed them with the nutrient solution at the ratio of oleic acid/palmitic acid (2:1, *v*/*v*) to make a 10 mM total FFA mixture, and then the mixture was filtered. The 10 mM total FFA mixture was diluted in a culture medium (1:10) to acquire the 1 mM final concentration. The cells were treated with 1 mM FFA for 24 h to induce fatty degeneration as a NAFLD cell model.

### 4.4. Measurement of Lipid and Peroxidation Levels

The cells were seeded in a six-well plate at a density of 1 × 10^5^ cells/well and were then cultured for 24 h. The cells were induced with FFA for 24 h and the old medium was discarded. The cells were treated with different concentrations of fucoxanthin (0.125, 0.25, 0.5, 1, 2, 4, and 8 µg/mL), which were dissolved in a culture medium without FFA for 24 h. Then, the cells were digested, centrifuged, and washed routinely with pre-chilled phosphate-buffered saline (PBS). We added 200 µL of Triton 100-X to each well and they were lysed on ice for 30 min. We then centrifuged at 3000 rpm for 10 min; collected the supernatant; and determined the levels of MDA, SOD, GSH-Px, CAT, TC, and TG in the cells using commercial kits, according to the manufacturer’s instructions.

### 4.5. ALT and AST Activity in Cells

The cells were seeded in a six-well plate at a density of 1 × 10^5^ cells/well, and after the treatment described above, each group of supernatant from the cell lysate in the six-well plates was taken as the sample to be measured [48]. The corresponding concentrations were measured using ALT and AST kits, respectively.

### 4.6. Measurement of Pro-Inflammatory Cytokines in Cells

According to the manufacturer’s instructions, an ELISA kit was used to quantify the levels of IL-1β, IL-6, and TNF-α in the cells. The expression values were calculated by bringing in a standard curve, and all data were repeated three times.

### 4.7. Oil Red O Staining

The cells were seeded in a six-well plate at a density of 1 × 10^5^ cells/well, and lipid droplets were produced according to the previously described method [48]. The cells were fixed at room temperature for 10 min by adding an appropriate amount of 10% paraformaldehyde, and were incubated with Oil Red O reagent for 15 min, and then re-stained with hematoxylin for 5 min and sealed. The accumulation of lipid droplets inside the cells was observed under a microscope, and they were photographed and recorded. Finally, the quantification of lipid droplets was analyzed using ImageJ software.

### 4.8. Western Blot Analysis

The cells were cultured in 1 mM FFA for 24 h as NAFLD model cells. After 24 h, the normal medium continued to incubate for 24 h. The normal group consisted of the cells cultured in a normal medium for 48 h. After the collected cells were lysed on ice with RIPA lysis buffer for 30 min, the supernatant was centrifuged and the protein concentration of each sample was determined using the BCA method. Then, we added an appropriate amount of 5× protein loading buffer and denatured the protein in a metal bath at 95 °C for 10 min, and stored it at −20 °C after sealing. It was then added to each well, after the electrophoresis proteins of different molecular weights were separated by SDS-PAGE and were transferred to polyvinylidene difluoride membranes. The detection was performed on the Fluor Chem FC3 system (Protein Simple, Waltham, MA, USA) using the enhanced chemiluminescence reagent (ECL, Solarbio, Beijing, China) (Figure S1). Image Lab software was used to analyze the protein levels, and β-actin was used as the internal control.

### 4.9. Statistical Analysis

Each treatment was carried out in triplicate, and data were expressed as mean ± standard deviation. Statistical results between experimental groups were analyzed by one-way ANOVA using SPSS 23.0 software. *p* < 0.05 indicates that the data are significantly different, and *p* < 0.01 indicates that the data are extremely significantly different, all of which are statistically significant. The results were subjected to the Brown−Forsythe analysis using GraphPad Prism 8.3.0, *p* ≥ 0.05 indicated that SDs were not significantly different (default *p* ≥ 0.05 without special markers).

## 5. Conclusions

In summary, fucoxanthin can alleviate oxidative stress, lipid accumulation, and inflammation in the NAFLD model of liver cells. Fucoxanthin treatment could reduce the concentration of oxidation products and the content of inflammatory factors, increase the activity of antioxidant enzymes, and then reverse the damage of the liver cell induced by FFA and reduce oxidative stress. Fucoxanthin treatment could also down-regulate the protein level of SREBP-1c and FAS, while activating the Nrf2 antioxidant signaling pathway and inhibiting the TLR4-mediated inflammation pathway, thereby protecting the cells.

## Data Availability

Not applicable.

## Supplementary Materials

The following supporting information can be downloaded at: https://www.mdpi.com/article/10.3390/md20040225/s1, Figure S1: Original image of Western blot.
