# Supplementary material for: Fucoxanthin Attenuates Free Fatty Acid-Induced Nonalcoholic Fatty Liver Disease by Regulating Lipid Metabolism/Oxidative Stress/Inflammation via the AMPK/Nrf2/TLR4 Signaling Pathway
Source: Mar Drugs. 2022 Mar 25;20(4):225. doi: 10.3390/md20040225 (PMC9027317; doi:10.3390/md20040225)

Figure S1: Original image of Western blot

The order of sample loading is from left to right: Control FFA L-Fx M-Fx H-Fx

p-AMPK

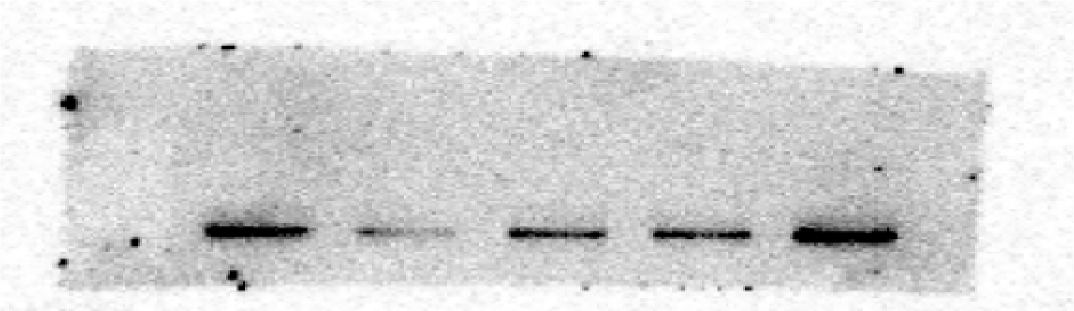

AMPK

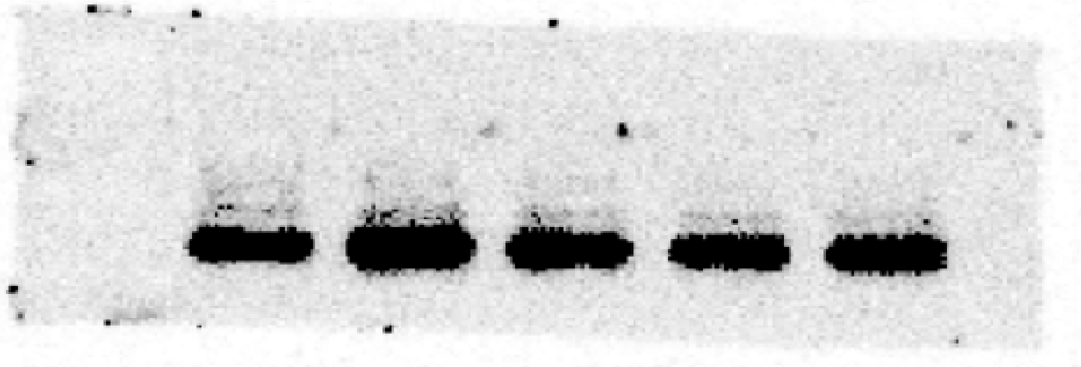

PPAR- α

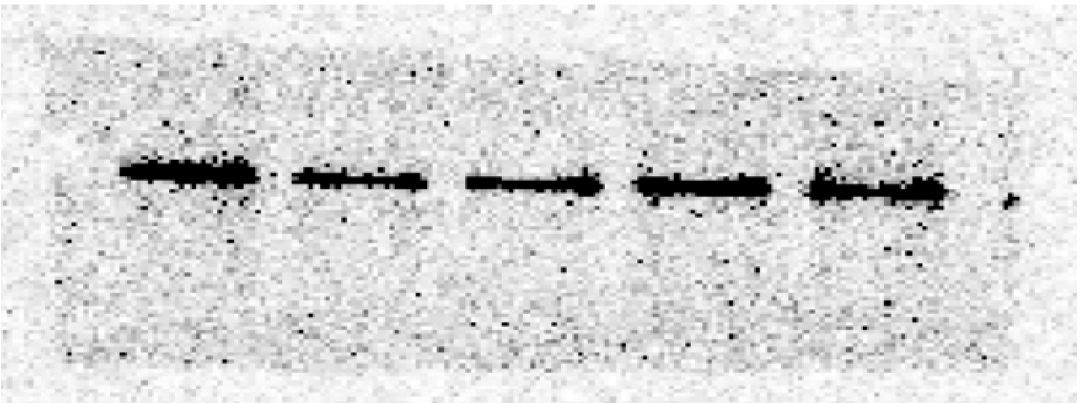

SREBP-1C

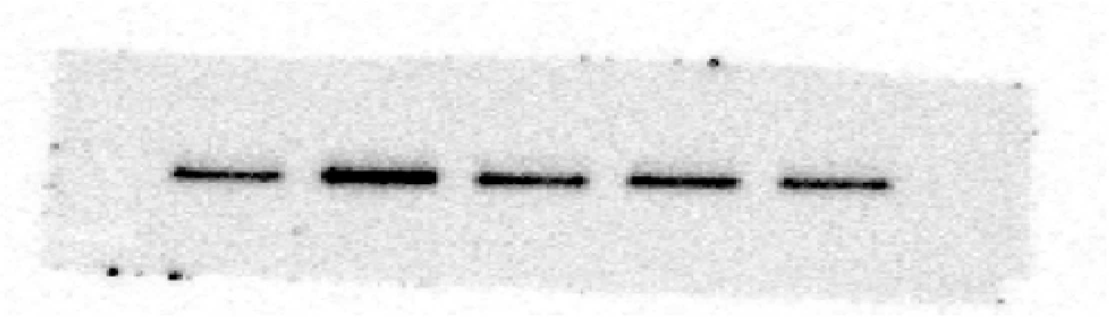

p-ACC

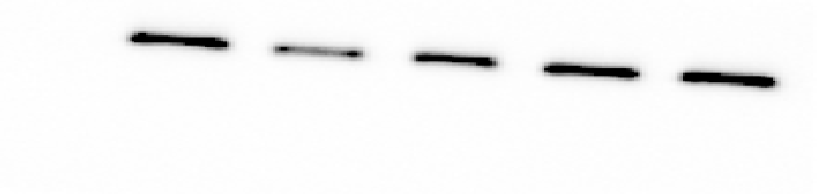

ACC

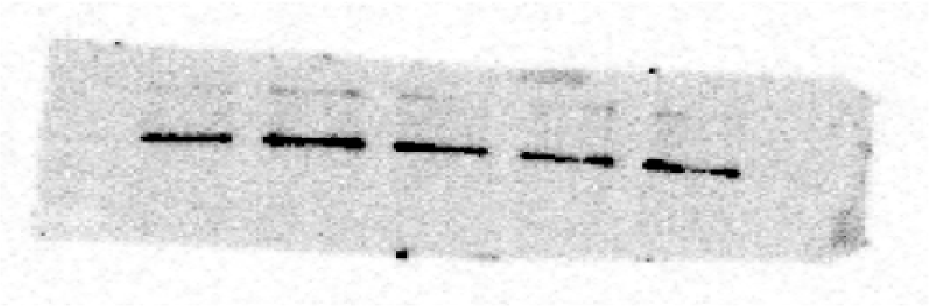

CPT-1

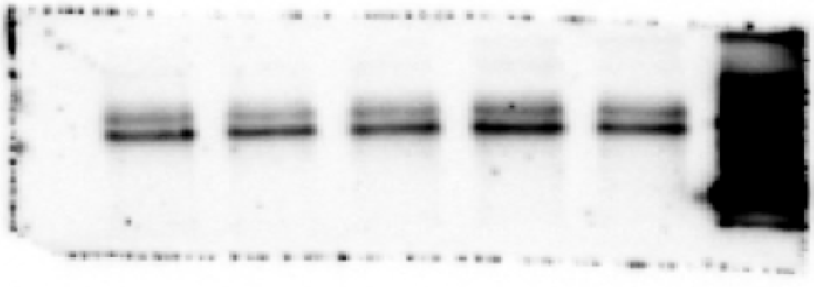

FAS

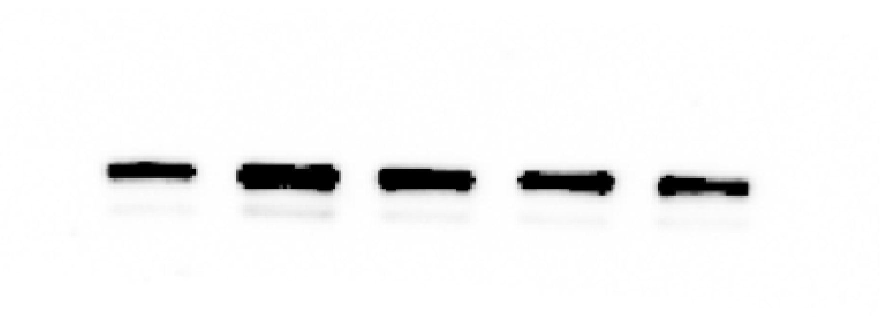

GAPDH-1

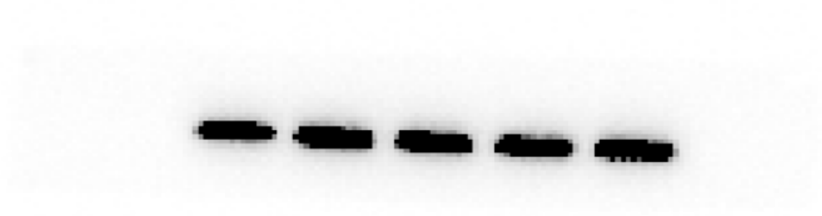

Keap1

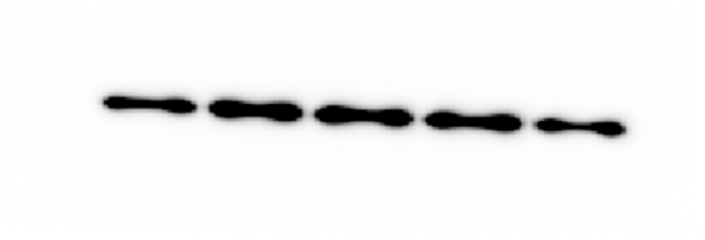

Nrf2

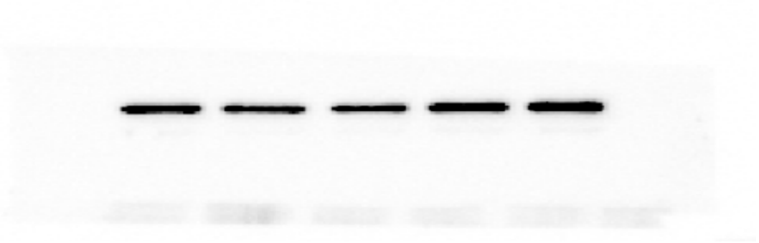

HO-1

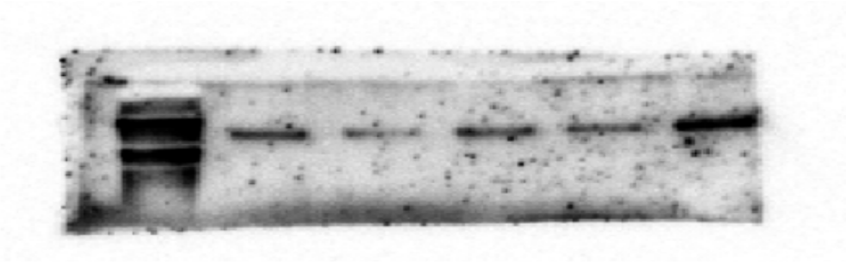

NQO1

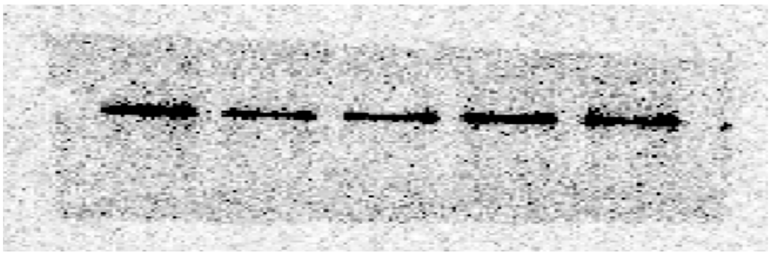

GCLM

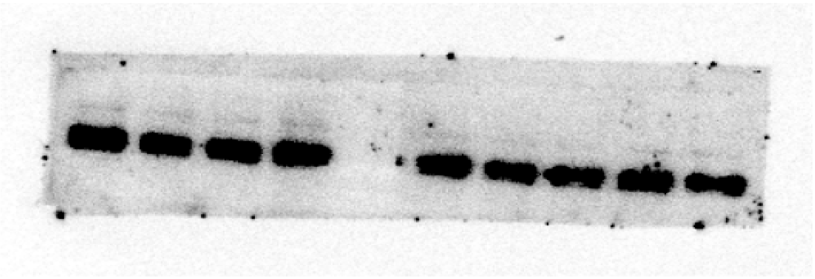

GAPDH-2

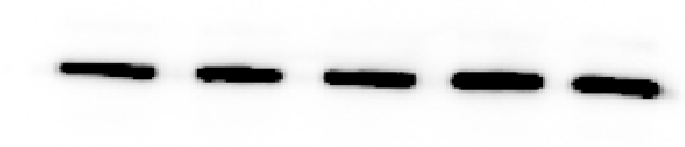

TLR4

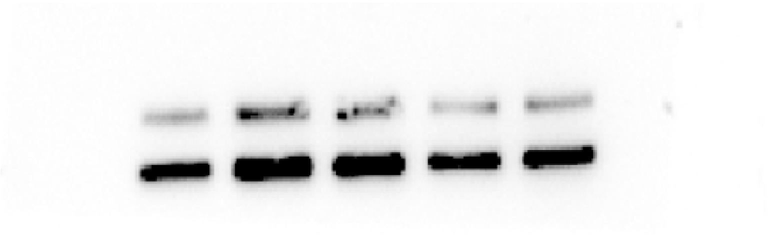

MyD88

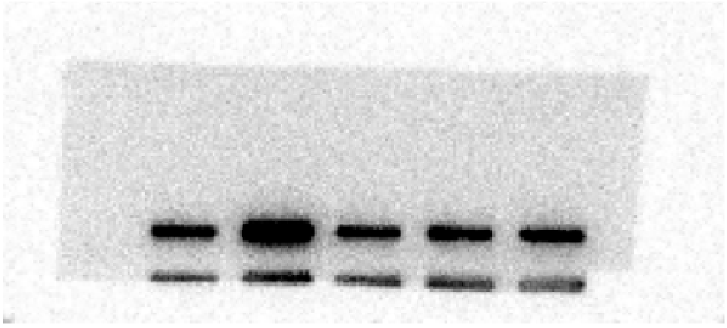

p-IκBα

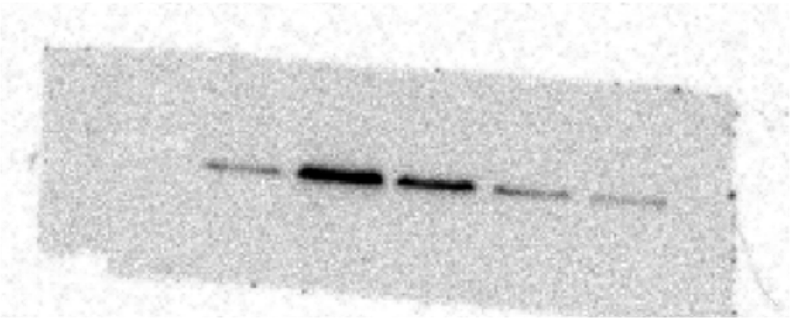

p-NF-kB p65

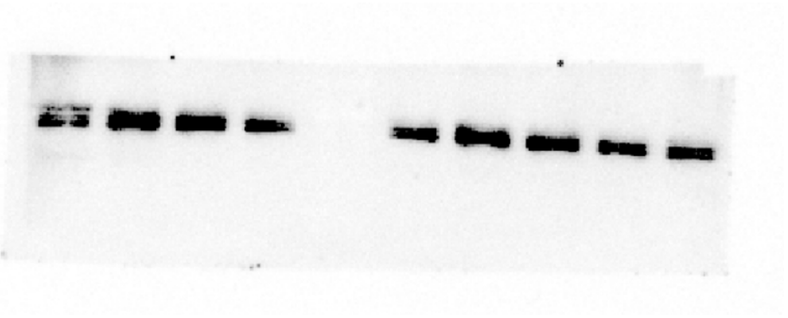

GAPDH-3

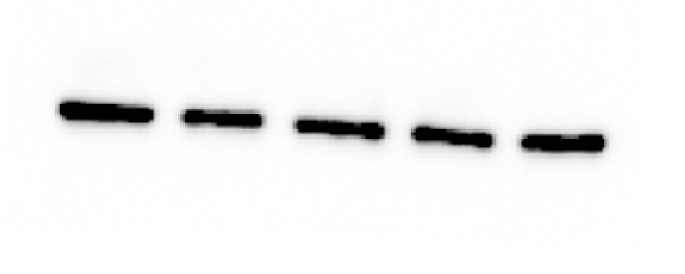

Supplement: Supplementary file 1 [file marinedrugs-20-00225-s001.zip › marinedrugs-1649843-supplementary.pdf]
